# Association between living environmental quality and risk of gastrointestinal diseases among middle-aged and older adults: evidence from a national study

**DOI:** 10.1186/s12889-026-28893-x

**Published:** 2026-08-04

**Authors:** Endian Liu, Yanbin Wei, Minjiao Wang, Yanqing Liu, Xin Yao, Jie Xing

**Affiliations:** 1 Department of Gastroenterology, Beijing Friendship Hospital, Capital Medical University, No.95 Yongan Road, Xicheng District, Beijing, 100050 P. R. China; 2 State Key Laboratory of Digestive Health, Beijing, P. R. China; 3 National Clinical Research Center for Digestive Disease, Beijing, P. R. China; 4 Beijing Key Laboratory of Early Gastrointestinal Cancer Medicine and Medical Devices, Beijing, P. R. China; 5 Department of Radiation Oncology, First Hospital of Shanxi Medical University, Taiyuan, P. R. China; 6 Department of Computer Science and Engineering, University of California, Santa Cruz, USA

**Keywords:** CHARLS, Living environmental quality, Gastrointestinal diseases, Middle-aged and older adults

## Abstract

**Background:**

With the rapid progression of population aging in China, gastrointestinal (GI) diseases have become one of the most common chronic conditions among middle-aged and older adults, substantially impairing quality of life and increasing healthcare burdens. However, evidence regarding the combined effects of multiple living environment factors and their potential interactions on GI health remains limited.

**Methods:**

This study used data from the China Health and Retirement Longitudinal Study from 2011 to 2020, including 10,032 middle-aged and older adults. The living environment quality (LEQ) was assessed based on building type, temperature comfort, water source, heating and cooking fuel, and PM_2.5_ exposure. Logistic regression and Cox proportional hazards models were applied to examine cross-sectional and longitudinal associations between LEQ and GI diseases.

**Results:**

The results showed that each one-point increase in LEQ score was associated with a higher risk of GI diseases in both cross-sectional analysis (OR = 1.05, 95% CI: 1.01–1.08, *P* = 0.013) and longitudinal analysis (HR = 1.06, 95% CI: 1.02–1.10, *P* < 0.001). Compared with the favorable LEQ group, the unfavorable LEQ group showed significantly higher odds and risks of GI diseases in cross-sectional (OR = 1.18, 95% CI: 1.03–1.35, *P* = 0.018) and longitudinal analyses (HR = 1.26, 95% CI: 1.10–1.45, *P* < 0.001), respectively, with a clear dose-response relationship. In longitudinal analyses, non-tap water and dual solid fuel for heating and cooking were significantly associated with an increased risk of GI diseases (all *P* < 0.05). Kaplan-Meier analysis further showed significant differences in the cumulative incidence of GI diseases across LEQ groups (log-rank *P* < 0.0001), with poorer LEQ associated with higher cumulative incidence. These associations remained robust in subgroup, interaction, and sensitivity analyses.

**Conclusions:**

Poorer LEQ may be associated with an increased risk of GI diseases among middle-aged and older adults, with non-tap water use and dual solid fuel use found as important environmental factors.

**Supplementary Information:**

The online version contains supplementary material available at 10.1186/s12889-026-28893-x.

## Background

With the rapid progression of population aging in China, adults aged 60 years and older accounted for approximately 17.8% of the total population in 2020. This proportion is expected to increase further [1]. The health of middle-aged and older adults has become a major public health concern [2]. Gastrointestinal (GI) diseases are among the most common chronic conditions in this population and represent a substantial health burden [3, 4]. Epidemiological evidence indicates that the incidence and prevalence of diarrheal diseases among adults aged 65 years and older have increased by nearly 200% globally, with China contributing substantially to this growing burden [5]. A global systematic review further reported that GI diseases are highly prevalent among older adults, with a considerably higher prevalence than that observed in the general adult population [6]. GI diseases can cause a wide range of symptoms, including abdominal pain, abdominal bloating, loss of appetite, and altered bowel habits, all of which can markedly impair quality of life. In addition, these conditions may interfere with nutrient digestion and absorption, thereby increasing the risks of malnutrition, frailty, cognitive impairment, and cardiovascular events [7, 8]. Persistent GI diseases may also progress to peptic ulcers and even GI malignancies, further increasing the health and economic burden on affected individuals and their families [9].

The living environment is the primary setting in which people spend most of their daily lives, and its quality has an important influence on health. Previous studies have shown that adverse living conditions, including household crowding, poor housing conditions, inadequate drinking water quality, and insufficient sanitation facilities, are associated with an increased risk of GI infectious diseases [5, 10]. In recent years, increasing attention has also been paid to the impact of outdoor environmental exposures on GI health, particularly fine particulate matter (PM_2.5_). A nationwide study further reported that long-term exposure to multiple air pollutants, including PM_2.5_, PM_10_, sulfur dioxide, and nitrogen dioxide, was significantly associated with an increased risk of GI diseases [11]. Furthermore, environmental factors such as ambient temperature and humidity may also contribute to the development and progression of GI diseases among middle-aged and older adults.

Although previous studies have confirmed the associations between certain living environmental factors and GI diseases, most studies focused on single environmental exposures, which could not comprehensively evaluate overall living environment quality (LEQ) [10, 12, 13]. Moreover, evidence regarding the combined effects of multiple living environment factors and their potential interactions on GI health remains limited. To address these gaps, this study used data from the China Health and Retirement Longitudinal Study (CHARLS) collected between 2011 and 2020 to systematically evaluate the association between LEQ and GI diseases among middle-aged and older adults. LEQ was assessed based on multiple dimensions of the living environment, including building type, temperature comfort, water source, heating and cooking fuel, and PM_2.5_ exposure. We further examined whether these associations differed across population subgroups. The findings may help identify key environmental risk factors and provide evidence to inform public health strategies for improving living environments and promoting GI health.

## Methods

### Study design

The data used in this study were obtained from the CHARLS [14]. CHARLS is a nationally representative cohort study of Chinese adults aged 45 years and older. The baseline survey was conducted in 2011, with follow-up surveys completed in 2013, 2015, 2018, and 2020. The study included participants from 150 counties (districts) and 450 communities (villages) across 28 provinces in China. CHARLS was conducted by the National School of Development at Peking University, and was approved by the Biomedical Ethics Review Committee of Peking University (No. IRB00001052-11015). All participants provided written informed consent before enrollment. The CHARLS data are publicly available through the CHARLS website (http://charls.pku.edu.cn/).

**Fig. 1:**
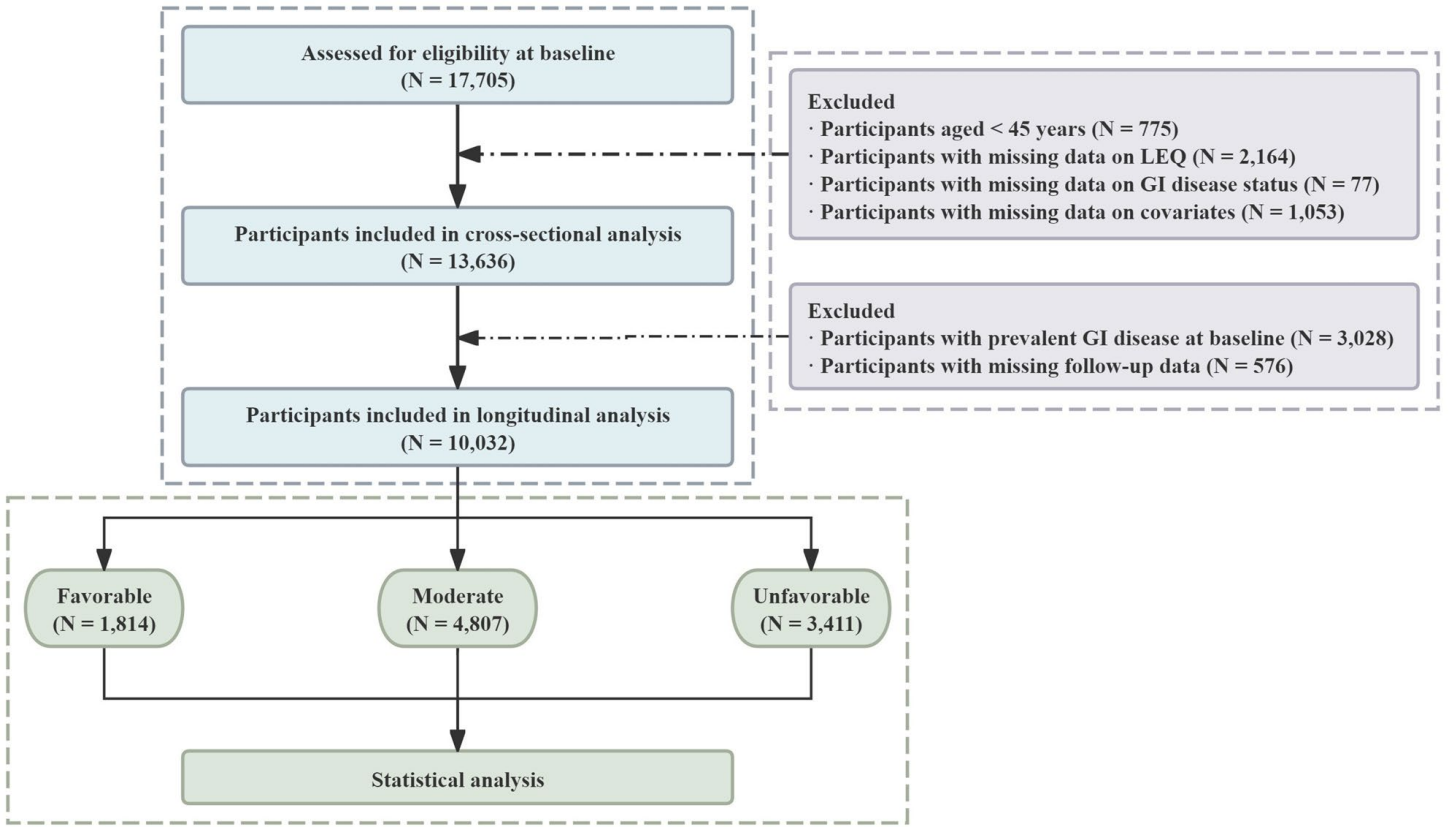
Flow diagram for participants included in the study. GI, gastrointestinal; LEQ, living environmental quality

This study used baseline data from the 2011–2012 CHARLS survey for the cross-sectional analysis of the association between LEQ and GI diseases. Participants were then followed through four follow-up surveys conducted between 2013 and 2020 for the longitudinal analysis. A total of 17,705 participants were initially enrolled in CHARLS. We excluded 4,069 participants who were younger than 45 years or had missing data on LEQ, GI diseases, or any covariates at baseline. The remaining 13,636 participants were included in the cross-sectional analysis. For the longitudinal analysis, we further excluded 3,028 participants with prevalent GI diseases at baseline and 576 participants without follow-up data. The final longitudinal analysis included 10,032 participants. The participant selection process is shown in Fig. 1.

### Assessments of LEQ

The LEQ was assessed based on five key indicators: building type, temperature comfort, water source, heating and cooking fuel, and PM_2.5_ exposure. The scoring criteria for each indicator are presented in Table 1. As there is currently no established theoretical or empirical evidence to support assigning different weights to individual environmental factors when constructing a composite LEQ index, an additive scoring approach was adopted. Specifically, multi-story building, comfortable indoor temperatures, tap water, and PM_2.5_ exposure < 35 µg/m^3^ were assigned a score of 0, whereas the corresponding unfavorable conditions were assigned a score of 1. Heating and cooking fuel were scored as 0, 1, or 2 according to the degree of solid fuel use. According to the Ambient Air Quality Standards issued by the Ministry of Ecology and Environment of the People’s Republic of China (https://www.mee.gov.cn/ywgz/fgbz/bz/bzwb/dqhjbh/dqhjzlbz/202602/W020260225340726552289.pdf), an annual PM_2.5_ concentration of 35 µg/m^3^ was used as the cutoff for high PM_2.5_ exposure. PM_2.5_ exposure data were obtained from the China High Air Pollutants dataset [15]. Due to restrictions on individual residential location information in the CHARLS database, city-level annual average PM2.5 concentrations were used as a proxy for individual exposure in this study. A composite LEQ score was calculated by summing the scores of all indicators, resulting in a total score ranging from 0 to 6. Higher scores indicated greater cumulative exposure to unfavorable living environments. Based on a previously established classification method, participants were categorized into three LEQ groups according to their LEQ scores: favorable (0–1), moderate (2–3), and unfavorable (4–6). This scoring approach was adopted from the original study that developed the LEQ index and has been used in subsequent studies, ensuring methodological consistency and comparability across studies [16–18].

**Table 1:** Components and scoring criteria of the LEQ index

Domain	Assessment	Classification	Score
Housing structure	Building type	Multi-story building	0
		One-story building	1
Indoor thermal comfort	Temperature comfort	Comfortable	0
		Uncomfortable	1
Drinking water quality	Water source	Tap water	0
		Non-tap water	1
Household energy use	Heating and cooking fuel	Clean fuel	0
		Mixed fuel	1
		Dual solid fuel	2
Ambient air pollution	PM_2.5_ exposure	< 35.0 µg/m^3^	0
		≥ 35.0 µg/m^3^	1
LEQ score	Sum of all component scores	Range: 0–6	Higher scores indicate poorer living environmental quality
LEQ classification	Environmental quality level	Favorable (0–1)	-
		Moderate (2–3)	-
		Unfavorable (4–6)	-

*Abbreviations: LEQ* Living environmental quality

### Assessments of GI diseases

GI diseases were defined based on self-reported physician diagnoses collected from the HEALTH STATUS AND FUNCTIONING questionnaire at baseline and each follow-up survey in CHARLS. Participants were asked, “Have you been diagnosed with stomach or other digestive disease (except for tumor or cancer) by a doctor?”. Participants who responded “yes” were classified as having GI diseases.

### Assessments of covariates

Based on previous studies and potential confounding factors, the following covariates were included in the analyses: age (years), gender (Female/Male), residence (Rural/Urban), marital status (Married and living with a spouse/Married but living without a spouse/Single, divorced, and widowed), education status (Elementary school or below/Middle school or above), smoking status (Non-smoker/Smoker), drinking status (Drink but less than once a month/Drink more than once a month/Non-drinker), region (Central/East/Northeast/West), and the number of multimorbidity (0/1/≥ 2) [11, 19]. The multimorbidity included: (1) hypertension; (2) dyslipidemia (elevation of low density lipoprotein, triglycerides, and total cholesterol, or a low high density lipoprotein level); (3) diabetes or high blood sugar; (4) cancer or malignant tumor (excluding minor skin cancers); (5) chronic lung diseases, such as chronic bronchitis, emphysema (excluding tumors, or cancer); (6) liver disease (except fatty liver, tumors, and cancer); (7) heart attack, coronary heart disease, angina, congestive heart failure, or other heart problems; (8) stroke; (9) kidney disease (except for tumor or cancer); (10) emotional, nervous, or psychiatric problems; (11) memory-related disease; (12) arthritis or rheumatism; and (13) asthma.

### Statistical analysis

Participants were stratified into three groups according to their baseline LEQ score. Continuous variables are presented as the mean and standard deviation, whereas categorical variables are presented as numbers and percentages. Differences in continuous variables among the three groups were assessed by one-way analysis of variance, and differences in categorical variables were compared by the chi-square test.

Cross-sectional associations were examined by logistic regression models based on baseline data collected in 2011–2012, with results reported as odds ratios (ORs) and 95% confidence intervals (CIs). Longitudinal associations were evaluated by Cox proportional hazards models with follow-up time as the underlying time scale, and results were reported as hazard ratios (HRs) and 95% CIs. The proportional hazards assumption was assessed by Schoenfeld residuals. Covariates that violated this assumption were handled with stratification. For participants who developed GI diseases during follow-up, time to event was defined as the interval from baseline to the first follow-up survey at which GI diseases were reported. For participants who remained free of GI diseases, follow-up time was defined as the interval from baseline to the last available follow-up survey. For both the cross-sectional and longitudinal analyses, three models with sequential adjustment for potential confounding factors were constructed. Model 1 was unadjusted. Model 2 was adjusted for age, gender, residence, marital status, education status, smoking status, drinking status, and region. Model 3 was further adjusted for the number of multimorbidity. Furthermore, Kaplan-Meier curves were performed to estimate the cumulative incidence of GI diseases across three LEQ groups, and differences between groups were assessed by the log-rank test.

Subgroup analyses were performed according to age, gender, residence, marital status, education status, smoking status, drinking status, region, and the number of multimorbidity. Potential effect modification was evaluated by including multiplicative interaction terms between LEQ and each subgroup variable. Several sensitivity analyses were conducted to evaluate the robustness of the findings: (1) restricting the analysis to participants with complete data on all covariates and outcome variables; (2) imputing missing covariate data using multiple imputation by chained equations and generating 50 imputed datasets [20]; (3) excluding participants who developed GI diseases during the first follow-up wave to minimize potential reverse causation and the influence of preclinical diseases at baseline.

All statistical analyses were performed using R software (version 4.2.2; R Foundation for Statistical Computing, Vienna, Austria). All tests were two-sided, and a *P* value < 0.05 was considered statistically significant.

## Results

### Characteristics of study population

A total of 10,032 eligible middle-aged and older adults were included in this study. According to the composite LEQ score, participants were classified into three groups: favorable LEQ (*N* = 1,814, 18.08%), moderate LEQ (*N* = 4,807, 47.92%), and unfavorable LEQ (*N* = 3,411, 34.00%) (Table 2). Compared with participants in the favorable LEQ group, those in the unfavorable LEQ group were older (*P* < 0.001), more likely to live in rural areas (*P* < 0.001), and had lower educational levels (*P* < 0.001). They also had higher proportions of being single, divorced, or widowed (*P* = 0.009), as well as higher proportions of smoking (*P* < 0.001) and drinking more than once per month (*P* < 0.001). In addition, participants with unfavorable LEQ were more likely to live in Western China (*P* < 0.001) and had a higher prevalence of multimorbidity (*P* = 0.035). No significant difference was observed in gender distribution among the three LEQ groups (*P* = 0.114).

**Table 2:** Baseline characteristics of participants according to LEQ

Characteristic	Overall	LEQ group			*P* value
		Favorable	Moderate	Unfavorable	
N (%)	10,032	1814 (18.08)	4807 (47.92)	3411 (34.00)	
Age [Mean (SD), years]	58.9 (9.6)	57.7 (9.5)	58.3 (9.5)	60.3 (9.6)	< 0.001^a^
Gender (n, %)					
Female	5210 (51.9)	974 (53.7)	2506 (52.1)	1730 (50.7)	0.114^b^
Male	4822 (48.1)	840 (46.3)	2301 (47.9)	1681 (49.3)	
Residence (n, %)					
Rural	6446 (64.3)	546 (30.1)	2964 (61.7)	2936 (86.1)	< 0.001^b^
Urban	3586 (35.7)	1268 (69.9)	1843 (38.3)	475 (13.9)	
Marital status (n, %)					
Married and living with a spouse	8141 (81.2)	1501 (82.7)	3924 (81.6)	2716 (79.6)	0.009^b^
Married but living without a spouse	602 (6.0)	113 (6.2)	285 (5.9)	204 (6.0)	
Single, divorced, and windowed	1289 (12.8)	200 (11.0)	598 (12.4)	491 (14.4)	
Education status (n, %)					
Elementary school or below	6973 (69.5)	979 (54.0)	3284 (68.3)	2710 (79.4)	< 0.001^b^
Middle school or above	3059 (30.5)	835 (46.0)	1523 (31.7)	701 (20.6)	
Smoking status (n, %)					
Non-smoker	6111 (60.9)	1221 (67.3)	2938 (61.1)	1952 (57.2)	< 0.001^b^
Smoker	3921 (39.1)	593 (32.7)	1869 (38.9)	1459 (42.8)	
Drinking status (n, %)					
Drink but less than once a month	863 (8.6)	173 (9.5)	403 (8.4)	287 (8.4)	< 0.001^b^
Drink more than once a month	2103 (21.0)	333 (18.4)	958 (19.9)	812 (23.8)	
Non-drinker	7066 (70.4)	1308 (72.1)	3446 (71.7)	2312 (67.8)	
Region (n, %)					
Central	2881 (28.7)	568 (31.3)	1401 (29.1)	912 (26.7)	< 0.001^b^
East	3261 (32.5)	662 (36.5)	1851 (38.5)	748 (21.9)	
Northeast	588 (5.9)	2 (0.1)	184 (3.8)	402 (11.8)	
West	3302 (32.9)	582 (32.1)	1371 (28.5)	1349 (39.5)	
Multimorbidity (n, %)					
0	2936 (29.3)	557 (30.7)	1448 (30.1)	931 (27.3)	0.035^b^
1	3283 (32.7)	592 (32.6)	1545 (32.1)	1146 (33.6)	
≥ 2	3813 (38.0)	665 (36.7)	1814 (37.7)	1334 (39.1)	

^a^One-way analysis of variance, ^b^Chi-square test

*Abbreviations*: *LEQ* Living environmental quality, *SD* Standard deviation

### Cross-sectional association between LEQ and GI diseases

The cross-sectional associations between LEQ and GI diseases are presented in Table 3. When LEQ was analyzed as a continuous variable, each 1-point increase in LEQ score was associated with higher odds of GI diseases (Model 1: OR = 1.08, 95% CI: 1.05–1.11, *P* < 0.001; Model 2: OR = 1.05, 95% CI: 1.01–1.09, *P* = 0.007; Model 3: OR = 1.05, 95% CI: 1.01–1.08, *P* = 0.013). When LEQ was categorized into three groups, participants in the unfavorable LEQ group had higher odds of GI diseases compared with the favorable LEQ group in three models (Model 1: OR = 1.34, 95% CI: 1.19–1.51, *P* < 0.001; Model 2: OR = 1.20, 95% CI: 1.05–1.38, *P* = 0.008; Model 3: OR = 1.18, 95% CI: 1.03–1.35, *P* = 0.018). Table S1 presents the specific environmental factors significantly associated with GI diseases, including the use of mixed fuel or dual solid fuel for heating and cooking (all *P* < 0.05). Building type, temperature comfort, water source, and PM_2.5_ exposure were not significantly associated with GI diseases after adjustment for potential confounding factors (all *P* > 0.05).

**Table 3:** Cross-sectional association between LEQ and GI diseases

Characteristic	*N*	Event (%)	Model 1		Model 2		Model 3	
			OR (95% CI)	*P* value	OR (95% CI)	*P* value	OR (95% CI)	*P* value
LEQ score	13,636	3028 (22.21%)	1.08 (1.05–1.11)	< 0.001	1.05 (1.01–1.09)	0.007	1.05 (1.01–1.08)	0.013
LEQ group								
Favorable	2425	465 (19.18%)	Ref		Ref		Ref	
Moderate	6490	1424 (21.94%)	1.18 (1.05–1.33)	0.004	1.13 (1.00-1.28)	0.051	1.11 (0.98–1.26)	0.090
Unfavorable	4721	1139 (24.13%)	1.34 (1.19–1.51)	< 0.001	1.20 (1.05–1.38)	0.008	1.18 (1.03–1.35)	0.018

Model 1 was the unadjusted model. Model 2 was adjusted for age, gender, residence, marital status, education status, smoking status, drinking status, and region. Model 3 was further adjusted for multimorbidity

*Abbreviations*: *CI* Confidence interval, *GI* Gastrointestinal, *LEQ* Living environmental quality, *OR* Odds ratio

### Longitudinal association between LEQ and GI diseases

Table 4 shows the longitudinal associations between LEQ and GI diseases. Consistent with the cross-sectional findings, continuous variable analysis showed that each 1-point increase in LEQ score was associated with a higher risk of incident GI diseases, which remained significant after sequential adjustment for potential confounders (Model 1: HR = 1.08, 95% CI: 1.05–1.12, *P* < 0.001; Model 2: HR = 1.06, 95% CI: 1.03–1.10, *P* < 0.001; Model 3: HR = 1.06, 95% CI: 1.02–1.10, *P* < 0.001). When LEQ was analyzed as a categorical variable, a clear dose-response relationship was observed. Compared with the favorable LEQ group, participants in the moderate (HR = 1.18, 95% CI: 1.04–1.33, *P* = 0.010) and unfavorable LEQ groups (HR = 1.26, 95% CI: 1.10–1.45, *P* < 0.001) had higher risks of developing GI diseases, and these associations remained significant in the fully adjusted Model 3. The associations between specific environmental factors and GI diseases are presented in Table S2. Non-tap water and dual solid fuel for heating and cooking were significantly associated with an increased risk of GI diseases in all models (all *P* < 0.05). In contrast, PM_2.5_ exposure ≥ 35 µg/m^3^ was associated with a lower risk of GI diseases in the fully adjusted model (HR = 0.87, 95% CI: 0.76–0.99, *P* = 0.04). Building type, temperature comfort, and the use of mixed fuel for heating and cooking were not significantly associated with GI diseases after adjustment for potential confounding factors (all *P* > 0.05).

**Table 4:** Longitudinal association between LEQ and GI diseases

Characteristic	*N*	Event (%)	Model 1		Model 2		Model 3	
			HR (95% CI)	*P* value	HR (95% CI)	*P* value	HR (95% CI)	*P* value
LEQ score	10,032	2349 (23.42%)	1.08 (1.05–1.12)	< 0.001	1.06 (1.03–1.10)	< 0.001	1.06 (1.02–1.10)	< 0.001
LEQ group								
Favorable	1814	349 (19.24%)	Ref		Ref		Ref	
Moderate	4807	1125 (23.4%)	1.21 (1.07–1.37)	0.002	1.19 (1.05–1.34)	0.007	1.18 (1.04–1.33)	0.010
Unfavorable	3411	875 (25.65%)	1.37 (1.21–1.55)	< 0.001	1.27 (1.11–1.46)	< 0.001	1.26 (1.10–1.45)	< 0.001

Model 1 was the unadjusted model. Model 2 was adjusted for age, gender, residence, marital status, education status, smoking status, drinking status, and region. Model 3 was further adjusted for multimorbidity

*Abbreviations*: *CI* Confidence interval, *GI* Gastrointestinal, *HR* Hazard ratio, *LEQ* Living environmental quality

The proportional hazards assumption was evaluated by Schoenfeld residuals. LEQ satisfied the proportional hazards assumption in both continuous and categorical analyses, whereas education status and region violated this assumption (*P* < 0.05) (Table S3). Therefore, stratified Cox proportional hazards models were applied for education status and region. The effect estimates from the stratified models were largely consistent with the main longitudinal analyses (Table S4), and the global Schoenfeld residual test confirmed that the proportional hazards assumption was no longer violated (Table S5).

### Cumulative incidence of GI diseases according to LEQ

**Fig. 2:**
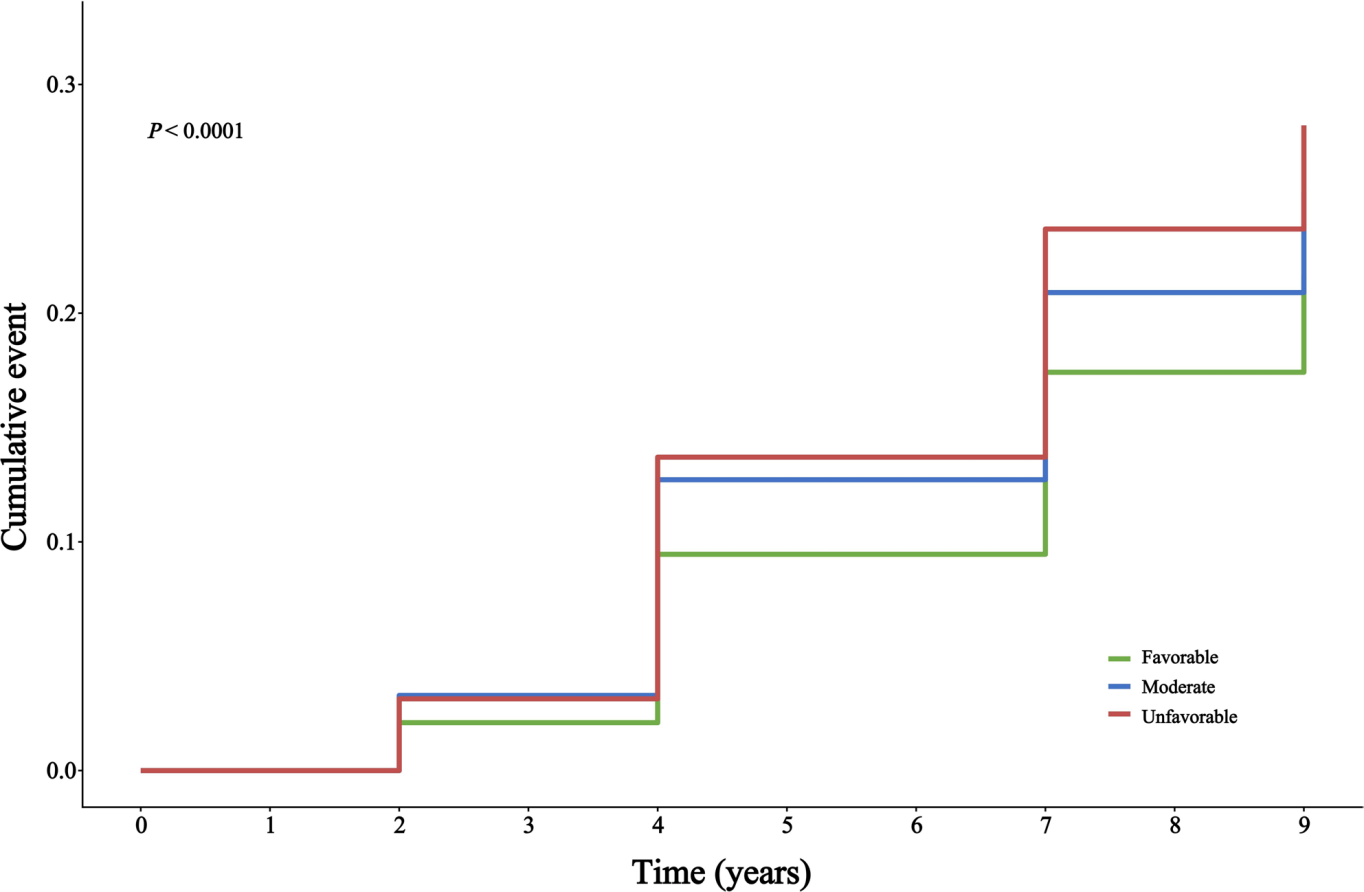
Cumulative incidence of GI diseases across different LEQ groups. The green, blue, and red lines represent the favorable, moderate, and unfavorable LEQ groups, respectively. GI, gastrointestinal; LEQ, living environmental quality

Figure 2 illustrates the Kaplan-Meier curves for the cumulative incidence of GI diseases across different LEQ groups. The log-rank test showed significant differences in cumulative incidence among the three groups (*P* < 0.0001). During follow-up, the cumulative incidence of GI diseases increased progressively in all groups. Participants in the unfavorable LEQ group consistently had the highest cumulative incidence, followed by those in the moderate LEQ group, whereas the favorable LEQ group showed the lowest incidence. These findings suggest a dose-response relationship between poorer LEQ and a higher incidence of GI diseases.

### Subgroup, interaction and sensitivity analyses

**Fig. 3:**
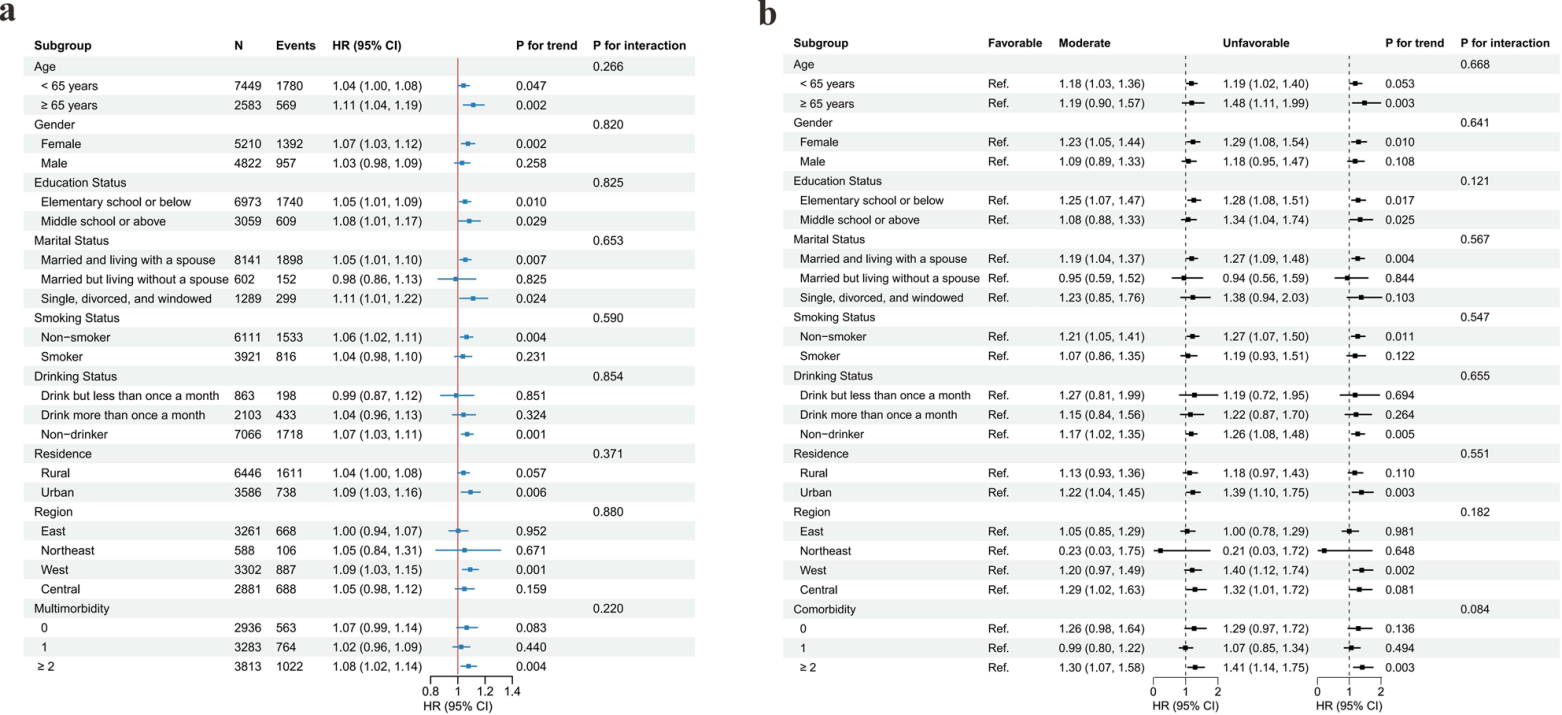
Association between LEQ and GI diseases in subgroups. **a** LEQ score, **b** LEQ group. GI, gastrointestinal; LEQ, living environmental quality

Figure 3 presents the results of subgroup analyses stratified by age, gender, education status, marital status, smoking status, drinking status, residence, region, and the number of multimorbidity. The association between LEQ and GI was generally consistent across all prespecified subgroups. No significant effect modification was observed for any stratification variable (all *P* values for interaction > 0.05).

Three sensitivity analyses were conducted to assess the robustness of the findings, including complete-case analysis, multiple imputation analysis, and exclusion of participants who developed GI diseases during the first follow-up wave (Table S6-S8). The complete-case analysis showed results generally consistent with the main analysis, although the associations were attenuated after adjustment for potential confounders, likely due to the reduced sample size (*N* = 5,879). Results based on multiple imputation datasets were highly consistent with the main findings, with significant associations observed across all models. After excluding early incident cases, the associations between LEQ and GI diseases remained similar to the primary analysis. Overall, the sensitivity analyses yielded consistent results, supporting the robustness of the main findings.

## Discussion

This study used data from a nationally representative sample of middle-aged and older adults in China to investigate the association between LEQ and GI diseases. We found that poorer LEQ could be associated with a higher risk of GI diseases, showing a consistent dose-response relationship. The association remained significant after adjustment for demographic characteristics, lifestyle factors, and comorbidities. Further analyses showed the use of solid fuels for heating and cooking as the important environmental risk factor, while non-tap water use was also associated with an increased risk of GI diseases. The findings remained robust across multiple statistical analyses. This study provides additional epidemiological evidence on the relationship between living environments and GI health and highlights the potential importance of improving living conditions to promote GI health among middle-aged and older adults.

Among the environmental factors examined, the use of solid fuels for heating and cooking was the important factor associated with GI diseases. Solid fuel combustion releases various harmful pollutants, including particulate matter, polycyclic aromatic hydrocarbons, volatile organic compounds, and heavy metals, which contribute to indoor air pollution and increase human exposure [21]. Consistent with our findings, a previous CHARLS-based cohort study reported that household solid fuel use, particularly for cooking, was associated with increased risks of GI and liver diseases [22]. The study further suggested that switching from solid fuels to clean fuels may reduce these health risks. In addition, households relying on solid fuels often have poor ventilation and lower socioeconomic status [23]. These factors may interact with pollutant exposure and further amplify the adverse health effects associated with solid fuel use.

Our study further found that non-tap water use was associated with an increased risk of GI diseases. Drinking water quality is an important environmental determinant of GI health. Compared with centrally treated tap water, untreated sources such as well water and river water are more susceptible to contamination by microbial pathogens, parasites, heavy metals, and organic pollutants, which may increase health risks. Previous studies have also supported this association. For example, Bradley et al. reported that contaminated drinking water was associated with adverse GI health outcomes in Massachusetts, USA [24]. Long-term exposure to contaminated water, particularly water with high levels of heavy metals or salts, may affect GI health through several mechanisms, including persistent immune activation, alterations in gut microbiota composition, and chronic inflammation [25–27]. And drinking water sources are closely related to local infrastructure and sanitation conditions [28]. Therefore, the observed association may also partly reflect broader environmental and socioeconomic disparities.

Notably, our longitudinal analysis showed that PM_2.5_ exposure ≥ 35 µg/m^3^ was associated with a lower risk of GI diseases, which contrasts with findings from most previous studies. Increasing evidence suggests that air pollution not only affects respiratory and cardiovascular health but may also impair GI health through the gut-lung axis [29, 30]. Experimental studies have shown that long-term PM_2.5_ exposure can alter gut microbiota composition and diversity, disrupt microbial metabolic functions, and affect intestinal homeostasis [31–33]. In addition, PM_2.5_ exposure may reduce the expression of tight junction proteins, such as occludin and ZO-1, leading to increased intestinal permeability, impaired barrier function, and activation of inflammatory pathways [32, 34, 35]. Consistent with these biological mechanisms, epidemiological studies have linked long-term PM_2.5_ exposure to increased risks of inflammatory bowel disease, functional GI disorders, and GI cancers [36]. Therefore, the inverse association observed in our study should be interpreted with caution. One possible explanation is exposure misclassification. Since PM_2.5_ exposure was estimated at the city level rather than measured at the individual level, it may not accurately represent participants’ long-term exposure. In addition, residual confounding and unmeasured factors cannot be completely excluded.

An important strength of this study is the use of a composite LEQ index rather than focusing on individual environmental exposures. Our findings suggest that the cumulative effects of multiple adverse environmental exposures may have a greater impact on GI health than any single exposure alone. In real-world settings, environmental risk factors such as air pollution, indoor pollution from solid fuel use, and contaminated drinking water often coexist. These factors may influence health through shared biological pathways, including oxidative stress, chronic inflammation, impaired intestinal barrier function, and gut microbiota dysbiosis. This finding is consistent with the exposome framework, which emphasizes that the combined effects of multiple environmental exposures across the life course may better explain disease risk than individual exposures considered separately [37]. The observed dose-response relationship in our study further supports this concept, as GI disease risk increased progressively with worsening LEQ. However, it should be noted that the observational design of this study supports only the identification of associations rather than causal relationships, particularly regarding the potential health benefits of improving LEQ.

This study has several limitations. Firstly, although CHARLS used a multistage stratified probability sampling design, survey weights and other complex sampling variables were not incorporated into our analyses. The generalizability of our findings to the broader Chinese population should be interpreted with caution. Secondly, CHARLS only collected information on whether participants had been diagnosed by a physician with “stomach or other digestive disease (except for tumor or cancer)” and did not provide detailed disease-specific information. Therefore, we were unable to assess the associations between LEQ and specific GI disease subtypes. Finally, GI disease status was assessed only during follow-up interviews, and the exact date of disease onset could not be determined, resulting in potential interval censoring of event times. This uncertainty in event timing may have reduced the precision of the estimated associations and potentially attenuated the observed effect estimates.

## Conclusions

Poorer LEQ may be associated with a higher risk of GI diseases among middle-aged and older adults. Non-tap water and dual solid fuel for heating and cooking were found as important environmental factors associated with GI diseases. Improving living conditions may help reduce the burden of GI diseases. Further studies are needed to confirm these associations.

## Supplementary Information

Supplementary Material 1.

## Data Availability

The datasets used and/or analysed during the current study are available from the corresponding author on reasonable request.

## Abbreviations

CHARLS: China Health and Retirement Longitudinal Study
CI: Confidence interval
GI: Gastrointestinal
HR: Hazard ratio
LEQ: Living environmental quality
OR: Odds ratio
SD: Standard deviation
