# Supplementary material for: Association between living environmental quality and risk of gastrointestinal diseases among middle-aged and older adults: evidence from a national study
Source: BMC Public Health. 2026 Aug 4;26:2343. doi: 10.1186/s12889-026-28893-x (PMC13466080; doi:10.1186/s12889-026-28893-x)
Supplement: Supplementary file 1 [file 12889_2026_28893_MOESM1_ESM.docx]

**Table S1** Cross-sectional association between living environmental factors and GI diseases

**Table S2** Longitudinal association between living environmental factors and GI diseases

**Table S3** Schoenfeld residual tests for proportional hazards assumption

**Table S4** Longitudinal association between LEQ and GI diseases in stratified Cox models

**Table S5** Schoenfeld residual tests for proportional hazards assumption after stratification

**Table S6** Sensitivity analyses of the longitudinal association between LEQ and GI diseases (using complete data)

**Table S7** Sensitivity analyses of the longitudinal association between LEQ and GI diseases (using multiple imputation data)

**Table S8** Sensitivity analyses of the longitudinal association between LEQ and GI diseases (excluding incident cases from the first follow-up wave)

| **Table S1** Cross-sectional association between living environmental factors and GI diseases | | | | | | | | | |
| --- | --- | --- | --- | --- | --- | --- | --- | --- | --- |
| **Characteristic** | **Model 1** | | | **Model 2** | | | **Model 3** | | |
|  | **OR (95% CI)** | ***P* value** | **OR (95% CI)** | | ***P* value** | **OR (95% CI)** | | ***P* value** |  |
| Building type |  |  |  | |  |  | |  |  |
| Multi-story building | Ref |  | Ref | |  | Ref | |  |  |
| Single-story building | 0.99 (0.91-1.08) | 0.773 | 0.94 (0.86-1.03) | | 0.197 | 0.93 (0.85-1.02) | | 0.130 |  |
| Temperature comfort |  |  |  | |  |  | |  |  |
| Comfortable | Ref |  | Ref | |  | Ref | |  |  |
| Uncomfortable | 1.07 (0.96-1.19) | 0.213 | 1.04 (0.93-1.16) | | 0.474 | 1.03 (0.92-1.14) | | 0.630 |  |
| Water source |  |  |  | |  |  | |  |  |
| Tap water | Ref |  | Ref | |  | Ref | |  |  |
| Non-tap water | 1.15 (1.06-1.25) | < 0.001 | 1.05 (0.96-1.14) | | 0.304 | 1.02 (0.94-1.12) | | 0.633 |  |
| Heating and cooking fuel |  |  |  | |  |  | |  |  |
| Clean fuel | Ref |  | Ref | |  | Ref | |  |  |
| Mixed fuel | 1.21 (1.10-1.33) | < 0.001 | 1.14 (1.03-1.27) | | 0.011 | 1.15 (1.04-1.28) | | 0.008 |  |
| Dual solid fuel | 1.35 (1.22-1.49) | < 0.001 | 1.24 (1.11-1.38) | | < 0.001 | 1.23 (1.10-1.38) | | < 0.001 |  |
| PM_2.5_ exposure |  |  |  | |  |  | |  |  |
| < 35 μg/m^3^ | Ref |  | Ref | |  | Ref | |  |  |
| ≥ 35 μg/m^3^ | 0.91 (0.80-1.03) | 0.128 | 1.05 (0.92-1.21) | | 0.476 | 1.11 (0.96-1.27) | | 0.160 |  |
| Model 1 was the unadjusted model. Model 2 was adjusted for age, gender, residence, marital status, education status, smoking status, drinking status, and region. Model 3 was further adjusted for multimorbidity. | | | | | | | | |  |
| *Abbreviations: CI, confidence interval; GI, gastrointestinal; OR, odds ratio.* | | | | | | | | |  |

| **Table S2** Longitudinal association between living environmental factors and GI diseases | | | | | | | | | |
| --- | --- | --- | --- | --- | --- | --- | --- | --- | --- |
| **Characteristic** | **Model 1** | | | **Model 2** | | | **Model 3** | | |
|  | **HR (95% CI)** | ***P* value** | **HR (95% CI)** | | ***P* value** | **HR (95% CI)** | | ***P* value** |  |
| Building type |  |  |  | |  |  | |  |  |
| Multi-story building | Ref |  | Ref | |  | Ref | |  |  |
| Single-story building | 1.05 (0.97-1.15) | 0.234 | 1.03 (0.95-1.13) | | 0.466 | 1.03 (0.94-1.13) | | 0.524 |  |
| Temperature comfort |  |  |  | |  |  | |  |  |
| Comfortable | Ref |  | Ref | |  | Ref | |  |  |
| Uncomfortable | 1.04 (0.93-1.16) | 0.46 | 1.01 (0.90-1.13) | | 0.875 | 0.99 (0.89-1.11) | | 0.899 |  |
| Water source |  |  |  | |  |  | |  |  |
| Tap water | Ref |  | Ref | |  | Ref | |  |  |
| Non-tap water | 1.23 (1.14-1.34) | < 0.001 | 1.14 (1.04-1.24) | | 0.003 | 1.13 (1.04-1.23) | | 0.006 |  |
| Heating and cooking fuel |  |  |  | |  |  | |  |  |
| Clean fuel | Ref |  | Ref | |  | Ref | |  |  |
| Mixed fuel | 1.17 (1.06-1.28) | 0.002 | 1.09 (0.99-1.21) | | 0.089 | 1.09 (0.99-1.21) | | 0.089 |  |
| Dual solid fuel | 1.36 (1.23-1.50) | < 0.001 | 1.26 (1.13-1.40) | | < 0.001 | 1.26 (1.13-1.40) | | < 0.001 |  |
| PM_2.5_ exposure |  |  |  | |  |  | |  |  |
| < 35 μg/m^3^ | Ref |  | Ref | |  | Ref | |  |  |
| ≥ 35 μg/m^3^ | 0.75 (0.66-0.84) | < 0.001 | 0.84 (0.74-0.96) | | 0.012 | 0.87 (0.76-0.99) | | 0.04 |  |
| Model 1 was the unadjusted model. Model 2 was adjusted for age, gender, residence, marital status, education status, smoking status, drinking status, and region. Model 3 was further adjusted for multimorbidity. | | | | | | | | |  |
| *Abbreviations: CI, confidence interval; GI, gastrointestinal; HR, hazard ratio.* | | | | | | | | |  |

| **Table S3** Schoenfeld residual tests for proportional hazards assumption | | | | | | | |
| --- | --- | --- | --- | --- | --- | --- | --- |
| **Model** | **Variable** | **LEQ score** | | | **LEQ group** | | |
|  |  | **Chi-square** | **df** | ***P* value** | **Chi-square** | **df** | ***P* value** |
| Model 1 |  |  |  |  |  |  |  |
|  | LEQ | 0.75 | 1 | 0.388 | 4.46 | 2 | 0.108 |
|  | GLOBAL | 0.75 | 1 | 0.388 | 4.46 | 2 | 0.108 |
| Model 2 |  |  |  |  |  |  |  |
|  | LEQ | 0.7 | 1 | 0.404 | 4.15 | 2 | 0.125 |
|  | Age | 0.02 | 1 | 0.896 | 0.02 | 1 | 0.895 |
|  | Gender | 1.76 | 1 | 0.184 | 1.76 | 1 | 0.185 |
|  | Education status | 6.36 | 1 | 0.012 | 6.33 | 1 | 0.012 |
|  | Marital status | 0.39 | 2 | 0.821 | 0.39 | 2 | 0.822 |
|  | Smoking status | 1.98 | 1 | 0.159 | 1.99 | 1 | 0.158 |
|  | Drinking status | 0.56 | 2 | 0.756 | 0.56 | 2 | 0.758 |
|  | Residence | 2.26 | 1 | 0.133 | 2.26 | 1 | 0.133 |
|  | Region | 10.02 | 3 | 0.018 | 9.98 | 3 | 0.019 |
|  | GLOBAL | 29.72 | 13 | 0.005 | 32.8 | 14 | 0.003 |
| Model 3 |  |  |  |  |  |  |  |
|  | LEQ | 0.69 | 1 | 0.407 | 4.09 | 2 | 0.129 |
|  | Age | 0.02 | 1 | 0.884 | 0.02 | 1 | 0.882 |
|  | Gender | 1.84 | 1 | 0.175 | 1.84 | 1 | 0.175 |
|  | Education status | 6.36 | 1 | 0.012 | 6.33 | 1 | 0.012 |
|  | Marital status | 0.38 | 2 | 0.827 | 0.38 | 2 | 0.829 |
|  | Smoking status | 1.88 | 1 | 0.170 | 1.89 | 1 | 0.169 |
|  | Drinking status | 0.58 | 2 | 0.749 | 0.57 | 2 | 0.751 |
|  | Residence | 2.23 | 1 | 0.135 | 2.24 | 1 | 0.134 |
|  | Region | 9.7 | 3 | 0.021 | 9.67 | 3 | 0.022 |
|  | Multimorbidity | 0.62 | 2 | 0.734 | 0.63 | 2 | 0.731 |
|  | GLOBAL | 30.03 | 15 | 0.012 | 33.14 | 16 | 0.007 |
| Chi-square statistics and corresponding *P* values are reported for each covariate and the global test. *P* < 0.05 indicates evidence against the proportional hazards assumption. | | | | | | | |
| *Abbreviations: LEQ, living environmental quality.* | | | | | | | |

| **Table S4** Longitudinal association between LEQ and GI diseases in stratified Cox models | | | | | | | | |
| --- | --- | --- | --- | --- | --- | --- | --- | --- |
| **Characteristic** | **N** | **Event (%)** | **Model 1** | | **Model 2** | | **Model 3** | |
|  |  |  | **HR (95% CI)** | ***P* value** | **HR (95% CI)** | ***P* value** | **HR (95% CI)** | ***P* value** |
| LEQ score | 10032 | 2349 (23.42%) | 1.08 (1.05-1.12) | < 0.001 | 1.06 (1.02-1.10) | < 0.001 | 1.06 (1.02-1.10) | 0.001 |
| LEQ group |  |  |  |  |  |  |  |  |
| Favorable | 1814 | 349 (19.24%) | Ref |  | Ref |  | Ref |  |
| Moderate | 4807 | 1125 (23.4%) | 1.21 (1.07-1.37) | 0.002 | 1.18 (1.04-1.34) | 0.008 | 1.18 (1.04-1.33) | 0.010 |
| Unfavorable | 3411 | 875 (25.65%) | 1.37 (1.21-1.55) | < 0.001 | 1.27 (1.11-1.46) | < 0.001 | 1.26 (1.10-1.45) | < 0.001 |
| Model 1 was unadjusted. Model 2 was adjusted for age, gender, marital status, smoking status, drinking status, and residence, and was stratified by education status (elementary school or below vs. middle school or above) and region (East, Central, Northeast, and West). Model 3 was further adjusted for multimorbidity and stratified using the same variables. | | | | | | | | |
| *Abbreviations: CI, confidence interval; GI, gastrointestinal; HR, hazard ratio; LEQ, living environmental quality.* | | | | | | | | |

| **Table S5** Schoenfeld residual tests for proportional hazards assumption after stratification | | | | | | | |
| --- | --- | --- | --- | --- | --- | --- | --- |
| **Model** | **Variable** | **LEQ score** | | | **LEQ group** | | |
|  |  | **Chi-square** | **df** | ***P* value** | **Chi-square** | **df** | ***P* value** |
| Model 1 |  |  |  |  |  |  |  |
|  | LEQ | 0.75 | 1 | 0.388 | 4.46 | 2 | 0.108 |
|  | GLOBAL | 0.75 | 1 | 0.388 | 4.46 | 2 | 0.108 |
| Model 2 |  |  |  |  |  |  |  |
|  | LEQ | 0.32 | 1 | 0.574 | 3.76 | 2 | 0.153 |
|  | Age | 0.47 | 1 | 0.494 | 0.46 | 1 | 0.497 |
|  | Gender | 0.74 | 1 | 0.390 | 0.73 | 1 | 0.392 |
|  | Marital status | 0.34 | 2 | 0.843 | 0.34 | 2 | 0.844 |
|  | Smoking status | 3.24 | 1 | 0.072 | 3.27 | 1 | 0.071 |
|  | Drinking status | 0.5 | 2 | 0.780 | 0.48 | 2 | 0.786 |
|  | Residence | 1.31 | 1 | 0.252 | 1.33 | 1 | 0.249 |
|  | GLOBAL | 14.27 | 9 | 0.113 | 17.2 | 10 | 0.070 |
| Model 3 |  |  |  |  |  |  |  |
|  | LEQ | 0.32 | 1 | 0.569 | 3.71 | 2 | 0.156 |
|  | Age | 0.42 | 1 | 0.515 | 0.42 | 1 | 0.519 |
|  | Gender | 0.79 | 1 | 0.373 | 0.79 | 1 | 0.375 |
|  | Marital status | 0.36 | 2 | 0.836 | 0.35 | 2 | 0.838 |
|  | Smoking status | 3.12 | 1 | 0.077 | 3.14 | 1 | 0.076 |
|  | Drinking status | 0.49 | 2 | 0.783 | 0.48 | 2 | 0.788 |
|  | Residence | 1.29 | 1 | 0.256 | 1.3 | 1 | 0.254 |
|  | Multimorbidity | 1.01 | 2 | 0.603 | 1.02 | 2 | 0.601 |
|  | GLOBAL | 14.97 | 11 | 0.184 | 17.92 | 12 | 0.118 |
| Chi-square statistics and corresponding *P* values are reported for each covariate and the global test. *P* < 0.05 indicates evidence against the proportional hazards assumption.  Model 1 was unadjusted. Model 2 was adjusted for age, gender, marital status, smoking status, drinking status, and residence. Model 3 was further adjusted for multimorbidity. Models 2 and 3 were stratified by education status (elementary school or below vs. middle school or above) and region (East, Central, Northeast, and West). | | | | | | | |
| *Abbreviations: LEQ, living environmental quality.* | | | | | | | |

| **Table S6** Sensitivity analyses of the longitudinal association between LEQ and GI diseases (using complete data) | | | | | | | | |
| --- | --- | --- | --- | --- | --- | --- | --- | --- |
| **Characteristic** | **N** | **Event (%)** | **Model 1** | | **Model 2** | | **Model 3** | |
|  |  |  | **HR (95% CI)** | ***P* value** | **HR (95% CI)** | ***P* value** | **HR (95% CI)** | ***P* value** |
| LEQ score | 5879 | 936 (15.92%) | 1.10 (1.04-1.15) | < 0.001 | 1.05 (1.00-1.11) | 0.068 | 1.05 (0.99-1.11) | 0.077 |
| LEQ group |  |  |  |  |  |  |  |  |
| Favorable | 972 | 128 (13.17%) | Ref |  | Ref |  | Ref |  |
| Moderate | 2914 | 449 (15.41%) | 1.19 (0.98-1.44) | 0.087 | 1.13 (0.92-1.39) | 0.232 | 1.12 (0.92-1.37) | 0.261 |
| Unfavorable | 1993 | 359 (18.01%) | 1.40 (1.15-1.72) | 0.001 | 1.22 (0.98-1.52) | 0.079 | 1.21 (0.97-1.51) | 0.089 |
| Model 1 was the unadjusted model. Model 2 was adjusted for age, gender, residence, marital status, education status, smoking status, drinking status, and region. Model 3 was further adjusted for multimorbidity. | | | | | | | | |
| *Abbreviations: CI, confidence interval; GI, gastrointestinal; HR, hazard ratio; LEQ, living environmental quality.* | | | | | | | | |

| **Table S7** Sensitivity analyses of the longitudinal association between LEQ and GI diseases (using multiple imputation data) | | | | | | | | |
| --- | --- | --- | --- | --- | --- | --- | --- | --- |
| **Characteristic** | **N** | **Event (%)** | **Model 1** | | **Model 2** | | **Model 3** | |
|  |  |  | **HR (95% CI)** | ***P* value** | **HR (95% CI)** | ***P* value** | **HR (95% CI)** | ***P* value** |
| LEQ score | 10834 | 2519 (23.25%) | 1.09 (1.06-1.12) | < 0.001 | 1.07 (1.03-1.11) | < 0.001 | 1.07 (1.03-1.10) | < 0.001 |
| LEQ group |  |  |  |  |  |  |  |  |
| Favorable | 1977 | 375 (18.97%) | Ref |  | Ref |  | Ref |  |
| Moderate | 5145 | 1196 (23.25%) | 1.22 (1.09-1.37) | < 0.001 | 1.21 (1.07-1.36) | 0.002 | 1.20 (1.06-1.35) | 0.003 |
| Unfavorable | 3712 | 948 (25.54%) | 1.39 (1.23-1.57) | < 0.001 | 1.31 (1.15-1.49) | < 0.001 | 1.30 (1.14-1.48) | < 0.001 |
| Model 1 was the unadjusted model. Model 2 was adjusted for age, gender, residence, marital status, education status, smoking status, drinking status, and region. Model 3 was further adjusted for multimorbidity. | | | | | | | | |
| *Abbreviations: CI, confidence interval; GI, gastrointestinal; HR, hazard ratio; LEQ, living environmental quality.* | | | | | | | | |

| **Table S8** Sensitivity analyses of the longitudinal association between LEQ and GI diseases (excluding incident cases from the first follow-up wave) | | | | | | | | |
| --- | --- | --- | --- | --- | --- | --- | --- | --- |
| **Characteristic** | **N** | **Event (%)** | **Model 1** | | **Model 2** | | **Model 3** | |
|  |  |  | **HR (95% CI)** | ***P* value** | **HR (95% CI)** | ***P* value** | **HR (95% CI)** | ***P* value** |
| LEQ score | 8423 | 1895 (22.5%) | 1.09 (1.05-1.13) | < 0.001 | 1.05 (1.01-1.09) | 0.012 | 1.05 (1.01-1.09) | 0.014 |
| LEQ group |  |  |  |  |  |  |  |  |
| Favorable | 1438 | 272 (18.92%) | Ref |  | Ref |  | Ref |  |
| Moderate | 4087 | 905 (22.14%) | 1.20 (1.04-1.37) | 0.010 | 1.14 (0.99-1.31) | 0.064 | 1.13 (0.98-1.30) | 0.084 |
| Unfavorable | 2898 | 718 (24.78%) | 1.37 (1.19-1.58) | < 0.001 | 1.21 (1.04-1.41) | 0.016 | 1.20 (1.03-1.39) | 0.020 |
| Model 1 was the unadjusted model. Model 2 was adjusted for age, gender, residence, marital status, education status, smoking status, drinking status, and region. Model 3 was further adjusted for multimorbidity. | | | | | | | | |
| *Abbreviations: CI, confidence interval; GI, gastrointestinal; HR, hazard ratio; LEQ, living environmental quality.* | | | | | | | | |
